# Attitudes toward death as a predictor of affective spectrum disorders in oncopsychology: a cross-cultural comparison

**DOI:** 10.1192/j.eurpsy.2025.1781

**Published:** 2025-08-26

**Authors:** K. Kholmuradova

**Affiliations:** Psychology, MSU, Tashkent, Uzbekistan

## Abstract

**Introduction:**

Affective spectrum disorders remain an underestimated problem in the field of oncopsychology, affecting not only the immune response and progression of some cancers in cancer patients (Smulevich, 2001; Reiche, Nunes, Morimoto, 2004; Schulz-Kindermann, 2021), but also performance and the provision of quality medical support by oncology staff (Mern, 2017; Klimenko, 2023). One of the possible factors contributing to depressive states and emotional burnout, we believe the peculiarities of attitudes toward death, which may have cross-cultural differences.

**Objectives:**

The main hypothesis of the study was the assumption that the nature of the attitude to death may be associated with both the level of depression in cancer patients and the severity of emotional burnout in medical workers of oncological institutions. We also assumed that this relationship may have cross-cultural differences due to differences in worldview attitudes (primarily related to the dominant type of religiosity), as well as due to organizational peculiarities of medical care in different countries.

**Methods:**

The study included 240 participants: 50 cancer patients from Russia and Germany (M_age_ = 55.3; SD = 8.6; 32 women and 18 men), and 190 medical specialists employed in oncologic institutions in Uzbekistan and Germany (M_age_= 37.1; SD = 9.6; 73 women and 117 men).

The study was based on a survey design using three questionnaires adapted in two languages (Russian and German): Beck’s Depression Inventory (Tarabrina, 1992; Hautzinger, Keller, Kühner, 2009), The Maslach Burnout Inventory (Vodopyanova, Starchenkova, 2001; Burisch, 2010), and Death Attitude Profile-Revised (Chistopolskaya, Mitina, Enikolopov, 2017; Jansen, Schulz-Quach, Eisenbeck, 2019). In addition, the study used questionnaires to collect data on the following characteristics of respondents.

**Results:**

It was revealed that the attitude towards death was found to be correlated both with the level of depression in cancer patients and with the syndrome of emotional burnout in workers of oncological institutions, namely: fear of death accompanies an increased level of affective disorders, while neutral acceptance - a lower level or none. Along with this, other variables were also identified that found a relationship with the development of affective spectrum disorders. Cross-cultural differences were also found regarding medical personnel: specialists employed in Uzbekistan showed a lower level of mental well-being, in contrast to personnel in Germany. When it comes to cancer patients, no culture-specific differences were found.

**Conclusions:**

According to the results of the study, it can be stated that the attitude to death can be not only a predictor of affective spectrum disorders (depression in cancer patients and emotional burnout in medical professionals), but also a resource in preventing these disorders. Neutral acceptance of death may be one of them.

**Disclosure of Interest:**

None Declared

